# Mary Sabine Taft

**Published:** 1910-03-15

**Authors:** 


MARY SABINE TAFT. Mrs. Taft was the widow of the late Dr. Johnathan Taft. She died after a lingering illness of over three years at Ann Arbor, January 13th, 1910. Mrs. Taft was Dr. Tafts second wife. She was a member of his household for many years previous to her marriage to Dr. Taft and knew most of the prominent members of the profession.

